# Neonatal Screening: Cost-utility Analysis for Galactosemia

**Published:** 2017-01

**Authors:** Nahid HATAM, Mehrdad ASKARIAN, Samad SHIRVANI, Elham SIAVASHI

**Affiliations:** 1. Dept. of Health Services Management, School of Management and Information Sciences, Shiraz University of Medical Sciences, Shiraz, Iran; 2. Dept. of Community Medicine, School of Medicine, Shiraz University of Medical Sciences, Shiraz, Iran; 3. Research Committee, Shiraz University of Medical Sciences, Shiraz, Iran

**Keywords:** Economic evaluation, Cost-utility analysis, Newborn screening, Galactosemia

## Abstract

**Background:**

Galactosemia is a congenital metabolic disorder that can damage the health of a newborn. Screening is an important step to prevent and treat this condition. Due to increasing health care costs and limited financial resources of health systems, the most suitable economic analysis tool should be applied. The aim of this study was to analyze the cost-utility of neonatal screening program for diagnosing galactosemia in Fars province, Iran.

**Methods:**

In this cross-sectional study and cost-utility analysis in the cost of screening for galactosemia and its financial effects, decision tree model and society’s viewpoint were used. The population of study was 81837 infants referred to Neonatal Screening Laboratory (Nader Kazemi Clinic) affiliated to Shiraz University of Medical Sciences (SUMS), Iran, in 2010. Quality of life in two groups of patients was evaluated by using the time trade-off. The best intervention option was selected by using the Incremental Cost-effectiveness Ratio.

**Results:**

The estimated cost of diagnosed through screening and without screening were 43519911 and 130011168 Iranian Rails (4222.00 $ and 12615.00 $), respectively. Therefore, there was a saving of 201443240.99 Iranian Rails (19641.00 $), for each patient annually.

**Conclusion:**

The screening program can improve both the qualitative and quantitative lifestyle of people and increase savings in health care system. Policymakers could use the results to design new policies based on the necessity of screening.

## Introduction

Genetic and congenital abnormalities are major factors in prenatal and neonatal mortalities (1). Each year, approximately 7.6 million children with severe congenital or genetic abnormalities are born; 90% of which occurs in low-income countries (2). Galactosemia is a genetic disorder with 25% chance of occurrence in the first-born child. This disease is caused by deficiency in galactose 1-phosphate enzyme that leads to metabolic disorder of galactose (3–6). Even though, its prevalence in Iran is relatively unknown but in Fars province it is 5:24000 (4,7).

Infants with this condition are usually born without any signs and symptoms. They will begin to emerge after breastfeeding (8, 9). The early symptoms include poor nutrition, jaundice, vomiting, and convulsion. In long-term, liver and kidney problems, cataracts, heart diseases, infections, neurological disorders, and ovarian dysfunction will appear (5, 10–13).

Early diagnosis in neonatal period, accompanied with galactose free diet can help to prevent long-term complications (14). Hence, the most important step in preventing and treating a patient suffering from metabolic disorders is its early diagnosis (15, 16). Through screening process, a patient is diagnosed with a simple and inexpensive test and in entered into treatment cycle (15).

World Health Organization (WHO) has emphasized on implementing preventive programs for genetic and congenital diseases in low-income and middle-income countries (17). Therefore, newborn screening should be considered as a priority in developing countries (18). Fortunately, neonatal screening is being implemented in most developed countries, while is growing in developing countries (19–21). According to each country condition, different tests are used (22). Neonatal screening for galactosemia is primarily aimed to diagnose clinical galactosemia (23). There are several tests available for it (19), and the simplest way is through urine (14).

Nowadays, many countries have delayed or ignored many important programs including screening (19). This is related to lack of resources, soaring health care costs and financing problem in health systems. Due to its important economic aspect, policymakers should pay more attention to allocate adequate budget for health services (24).

Therefore, selecting an appropriate method with high level of effectiveness and lower cost is essential (25). The cost-utility analysis is one of the most appropriate techniques for economic evaluation; it considers life expectancy as well as quality of life. Use of these techniques in health-care domain can provide an alternative solution for policy-makers (1, 26, 27).

To implement a program in macro level when there is no evidence available, at first a pilot project should be conducted, to be analyzed and then applied (28). In 2004, neonatal screening program was launched in Iran (29). Unfortunately, there is no confirmed study on the subject of galactosemia screening cost-utility in Iran. Hence, we have decided to analyze it by comparing cost per unit of utility in the city of Shiraz, Iran in 2010.

## Materials & Methods

This is a cross-sectional and an economic evaluation study. The population of study was 81837 infants referred to Neonatal Screening Laboratory (Nader Kazemi Clinic) affiliated to Shiraz University of Medical Sciences (SUMS), Iran, in 2010.

**Fig. 1:**
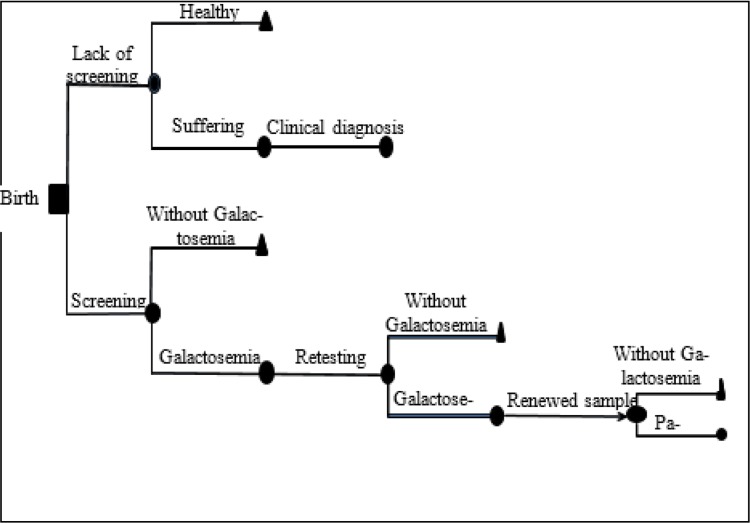
The decision tree of neonatal screening program for galactosemia

We applied the decision tree model. The first step was to define accurately our objective. The next step was to increase the population by determining the possibilities. Finally, costs and outputs of each path were estimated. Fig. 1 shows the decision tree model for galactosemia screening.

### Cost analysis

Data was collected based on society’s viewpoint. This viewpoint determines costs should be taken into account. Based on patient’s viewpoint, we have just calculated only the direct costs. Other indirect and intangible costs were not taken into consideration.

Thus, only direct costs of SUMS, insurance organizations, charities and costs that a patient has to provide for treatment through screening process have been considered, similarly, direct costs of treatment for those who did not go through screening process have been included. Then costs in both groups were compared.

Treatment costs of neonates whose test results were positive include; laboratory and clinical test, baby formula, visiting physician, wages, special diet, dentistry, medicine, paraclinical tests, as well as the cost of screening. The costs of screening are, running and capital costs. Running costs include; personnel costs, disposable goods, maintenance of buildings and facilities, and postage cost for sample transfer. In addition, capital costs include; depreciation of public and private facilities.

The total cost of care for galactosemia patients who did not go through screening process was calculated. To do so, medical documents, physicians’ comments, estimated number of admission per year, and the list of received services was analyzed. Then, the tariffs of private sector (after deduction of profit) were used in order to calculate the cost of each service package.

### Utility analysis

In order to calculate utility, time trade-off method was applied. In this method, interviewees had to choose between two health patterns: one is longer life expands with more health complications and the other is a shorter life expand but better quality of life. They were asked to choose between either life expand or quality of life (26). To assess the utility, interviews were conducted with nurses in charge of these patients, since they were more aware of their condition. Nurses were selected according to their experience and knowledge of the hospitalized patients. Sample size was 36 nurses with 95% probability, a standard deviation of 3 and minimum error difference of 2 using the following formula:

$$N=\frac{{\left(Z_{1-\alpha /2}+Z_{1-\beta }\right)}^{2}\times S^{2}}{d^{2}}$$

(N= desired sample size, α= type I error, β= type II error, d= difference between population and sample mean values)

Simple random sampling method using a table of random numbers was used. After selecting the samples and obtaining written informed consent, 36 nurses were interviewed. In these interviews, the two versions of forms (one for patients received screening and treatment and the other for patients suffering from the disease) were prepared. In both forms, a description of the disease and the patient’s status, the treatment method and diet were mentioned. The interviewees were asked whether they preferred to live 10 yr longer with critical a condition that ultimately leads to death or living less but with a better health condition.

To present cost-effectiveness analysis and to compare different intervention options Incremental Cost-Effectiveness Ratio was used (ICER).

After calculating the implementation cost of neonatal screening and the costs of treating people diagnosed through screening process, these costs summed up and deducted from the cost of treating galactosemia patients. Then, the result was divided by QALYs (Quality-Adjusted Life Year) gained from time trade-off. The obtained figure is ICER.

Given that, the benefits and results of neonatal screening will prevail over time, to calculate the present value of screening results (higher life expectancy and quality); future costs of treatment and future quality of life were reduced, by using 3% discount rate.

### Sensitivity analysis

The sensitivity analysis was used in order to reduce the impact of bias or measurement errors. To determine the acceptable range of parameters in this study, two methods were used. At first, 20% cost variables including; discount rates, prevalence rates and life expectancy were added and subtracted from each variable. For utility parameter, the confidence interval was calculated at 95% probability and was added to and subtracted from the mean; therefore, that the top and bottom range for utility and cost was determined. Afterward, one-way and two-way sensitivity analyses were performed. To present the results of one-way sensitivity analysis, the index of “Net Monetary Benefit” (NMB) was used and for twoway sensitivity analysis the “Worst–Best Analysis” (WBA) was used.

## Results

To implement neonatal-screening program, all newborn in the province were screened for diseases such as galactosemia, phenylketonuria, congenital hypothyroidism, and favism. The cost of screening and treatment for 81837 newborn, referred to the neonatal screening laboratory of SUMS, were calculated (Table 1).

**Table 1:** The cost of infants screening and treatment at SUMS, 2010

**costs of galactosemia**	**Costs (Iranian Rails)**	Costs (US dollars*)
Cost of neonatal screening program	811,088,637	78,703
Costs of galactosemia treatment (with screening)	43,519,911	4,222
Costs of galactosemia treatment (without screening)	130,011,168	12,615

* Average rate per dollar was 10,305.66 Iranian Rails in 2011 (30)

The cost of galactosemia screening for 81837 infant was 811088637 Iranian Rails ($ 78,703), and 9911 Iranian Rails per person. Furthermore, the tariff approved by the Ministry of Health and Medical Education for galactosemia screening in 2010 was 5600 Iranian Rails. In fact, the total cost of screening was 64846 Iranian Rails and the ratio for galactosemia was 7.42 percent (4811 Iranian Rails) of the total cost. Hence, the differentiation in price is spent on the treatment of patients and follow-ups.

If the result of screening was positive, the patient was referred to an appropriate clinic for treatment and follow-ups. From 81837 newborn 47 cases were diagnosed with galactosemia. The cost of treating these infants in the first three years of their lives is described in Table 2.

**Table 2:** Galactosemia treatment costs in first three years of life

**Years of life**	**Costs of treatment (Iranian Rails)**	**Cost (US Dollars)**
Galactosemia treatment costs in the first year of life	43519911	4222
Galactosemia treatment costs in the second year of life	43510000	4221
Galactosemia treatment costs in the third year of life	14610000	1417

Based on table 2, the maximum cost of treating galactosemia infants belongs to the first year of life (43519911 Iranian Rails).

The cost of treating galactosemia patients was estimated at 130011168 Iranian Rails. The mentioned cost is related to their life expand calculated with the discount rate for the future. To calculate the utility, direct time trade-off method was used. The results of time trade-off method and measuring quality of life in both screened and non-screen modes are presented in Table 3.

**Table 3:** Results of executing time tradeoff for galactosemia patients at SUMS, 2010

**Terms of screening**	**Without screening**	**With screening**
The number of nurses interviewed	36	36
Mean of utility	0.4750	0.8958
Standard Deviation	0.2771	0.1513
Maximum	1	1
Minimum	0	0.4

Based on Table 3, the utility of galactosemia without screening process was 0.4750, but for those who went through the screening process the utility was upgraded to 0.8958 close to 1.

Moreover, the results from economic evaluation of neonatal screening for galactosemia showed that the ratio of ICER was 201443240.99 ($ 19641.00).

Incremental cost-effectiveness ratio showed that the screening for galactosemia is predominant and is more cost-effective than not being screened. Not only the cost of screening was lower than treatment costs without being screened, however, quality of life in the screened patients was better. Hence, when screened total saving was 201443240.99 Iranian Rails ($ 19,641.00) per patient.

To display the results of one-way sensitivity analysis and the effect of each variable on the outcome of the program, the NMB index was equal to 202170.7763.

Due to positive value of NMB, the screening cost for galactosemia was less than its benefits (QALYs achieved through screening).

Then, multi-way sensitivity analysis was performed in WBA way. The results are shown in Table 4.

**Table 4:** Two-way sensitivity analysis to show fluctuations of costs and utility in galactosemia screening at SUMS, 2010

**Utility Cost**	**Minimum utility**	**Average utility**	**Maximum utility**
Minimum cost	366115598.15	−260333167	−201975567.14 The best scenario
Average cost	−282782675.76	−201077774.14	−156003162.95
Maximum cost	−199449752.25 The worst scenario	−141822380.48	−110030758.13

Multi-way sensitivity analysis showed that screening in the event of the worst scenario could save 199449752.25 Iranian Rails ($ 19,353.41) per galactosemia patient and in the event of the best scenario; the saving could be 201975567.14 Iranian Rails ($ 19598.5) per galactosemia patient. Furthermore, based on the results of one-way and two-way sensitivity analyses, the results are valid.

## Discussion

This study aimed to analyze the cost-utility of neonatal screening program and non-screening treatment of galactosemia patients.

The results showed that by implementing screening program, the cost of treatment is reduced to about one-third, compared to non-screening treatment.

The average utilities of screening and non-screening treatments using the time trade-off method were 0.896 and 0.475, respectively. This means that the utility will almost double through screening. Screening process health of galactosemia patients improved by 1.33 times, in comparison to its costs, therefore, implementing a screening program is cost-effective (27) which is consistent with our study.

ICER in our study showed that through the implementation of screening, 201443240.99 Iranian Rails ($ 19,641) could be saved per patient.

Based on our findings of one-way sensitivity analysis, the cost of screening for galactosemia is less than the achieved benefits (QALYs obtained from screening), and consequently, implementation of screening is cost-effective.

The ICER of screening compared with the lack of screening was approximate $12000 per QALY. That is the screened population benefited from QALY more than the unscreened population. Therefore, the neonatal screening in Texas was cost-effective compared with lack of screening (31) which is also consistent with our study.

Furthermore, the annual cost of neonatal screening for galactosemia was $489749.95. There were economic advantages for neonatal screening in comparison with lack of screening. The benefit will increase by reducing the economic interest rate (32).

The differences between incremental effectiveness in the case of implementation of screening and non-screening were 0.00005, which means an increase in each QALY. Moreover, the ratio of incremental cost-effectiveness (cost obtained per QALY) was $ 94,000. If it reaches $ 90000 per QALY, screening for galactosemia is no longer economical. Initial diagnosis of galactosemia can prevent infants’ deaths; however, by doing so the lifetime costs goes up due to complications (33). Hence, the result of this study is inconsistent with ours. This could be related to several factors such as differences in the structure of research, cost approach, and context of each country, which can be problematic for international generalization (34).

In addition, one of the criteria accepted by Wilson and Jungner to incorporate a test for the screening program is its positive cost-benefit (35). Cost-benefit analysis of screening for galactosemia was 0.2 and this value could increase when it is combined with other diseases (36). This outcome increases the acceptability of galactosemia screening.

There are several barriers to screening such as the society’s attitude towards the disease (37), costs, availability of diagnostic and therapeutic facilities, staff training, living in remote areas, difficulty in follow-ups, political and financial commitment from ministry of health to provide screening and management of the disease (37, 38), prevalence of the disease (38–40), national priorities, and costs versus benefits of screening and treatment (38).

However, factors such as screening quality by reducing the number of false positive results can reduce costs (33), the use of health care information systems for neonatal screening (40), the use of inexpensive diagnostic techniques for developing countries that have limited resources (19), proper organization of logistics for screening, establishing a centralized laboratory (22), and the combination of different tests in one screening program can play a significant role in increasing the cost-effectiveness of screening program. Finally, if we go one-step back, i.e. provide families with genetic counseling, it is possible to reduce national burden of genetic disorders.

There were two limitations in this study; the benefits of screening only calculated for the patients, but if the family benefits were also included, the effectiveness of screening program would increase dramatically. Another limitation was that we merely had access to data provided by SUMS. Therefore, we were forced to conduct our study in the city of Shiraz, Iran.

## Conclusion

Screening program is both socially acceptable and cost-effective. With respect to costs saving issue as well as increase in patient’s quality of life, those who participated in the screening program it seems that prolonging the program is feasible. Further studies could examine the cost-utility of screening program for different kind of diseases by considering indirect and intangible costs as well. This could be a starting point for policymakers to initiate extensive screening programs that can help to reduce cost and improve quality of life.

## Ethical considerations

Ethical issues (Including plagiarism, informed consent, misconduct, data fabrication and/or falsification, double publication and/or submission, redundancy, etc.) have been completely observed by the authors.

## References

[1] Hajizade M (2008). Health Economic. First ed Tehran: Jamenegar publication. Tehran.

[2] Report by the Secretariat (2005). Control of genetic diseases. World Health Organization. http://apps.who.int/iris/bitstream/10665/20404/1/B116_3-en.pdf?ua=1.

[3] Ficicioglu C Thomas N Yager C Gallagher PR Hussa C Mattie A Day-Salvatore DL Forbes BJ (2008). Duarte (DG) galactosemia: A pilot study of biochemical and neurodevelopmental assessment in children detected by newborn screening. Mol Genet Metab, 95 ( 4): 206–12. 18976948 10.1016/j.ymgme.2008.09.005

[4] Mirzajani F Mirfakhraei R Saki S Houshman M Naghibzadeh N Nabati F Talachian E (2005). Biochemical diagnosis and identification of common mutations in Galactosemia. Quarterly J Rehab, 6 ( 1): 19–22.

[5] Vakili R Rasooli Sh (2002). Glucose-Galactose Malabsorption as a Rare Cause of Diarrhea in Newborn Period. Iran J Pediatr, 12 ( 4): 20–22.

[6] Shield JP Wadsworth EJ MacDonald A Stephenson A Tyfield L Holton JB Marlow N (2000). The relationship of genotype to cognitive outcome in galactosaemia. Arch Dis Child, 83 ( 3): 248–50. 10952646 10.1136/adc.83.3.248 PMC1718484

[7] Senemar S Ganjekarimi A Senemar S Tarami B Bazrgar M (2011). The prevalence and clinical study of galactosemia disease in a pilot screening program of neonates, southern iran. Iran J Public Health, 40 ( 4): 99–104. 23113108 PMC3481732

[8] Wang BB Xu YK Ng WG Wong LJ (1998). Molecular and biochemical basis of galactosemia. Mol Genet Metab, 63 ( 4): 263–69. 9635294 10.1006/mgme.1998.2678

[9] Elsas LJ Lai K (1998). The molecular biology of galactosemia. Genet Med, 1 ( 1): 40–8. 11261429 10.1097/00125817-199811000-00009

[10] Estrada SC Canson DM Silao CL (2013). Mutational Analysis of the GALT Gene in Filipino Patients. Kobe J Med Sci, 59 ( 3): E106–11. 24045215

[11] Takci S Kadayifcilar S aCoskun T Yigit S Hismi B (2012). A Rare Galactosemia Complication: Vitreous Hemorrhage. JIMD Rep, 5: 89–93. 23430922 10.1007/8904_2011_103 PMC3509908

[12] Najafi M Khodadad A Khatami GR (2005). Cerebral edema: a rare complication in Galactosemia. Med J Islam Repub Iran, 18 ( 4): 371–73.

[13] van Erven B Gubbels CS van Golde RJ Dunselman GA Derhaag JG de Wert G Geraedts JP Bosch AM Treacy EP Welt CK Berry GT Rubio-Gozalbo ME (2013). Fertility preservation in female classic galactosemia patients. Orphanet J Rare Dis, 8: 107. 23866841 10.1186/1750-1172-8-107 PMC3718676

[14] Jensen UG Brandt NJ Christensen E Skovby F Nørgaard-Pedersen B Simonsen H (2001). Neonatal Screening for Galactosemia by Quantitative Analysis of Hexose Monophosphates Using Tandem Mass Spectrometry: A Retrospective Study. Clin Chem, 47 ( 8): 1364–72. 11468223

[15] Ordookhani A Hedayati M Mirmiran P Hajipour R Azizi F (2000). High prevalence of neonatal hypothyroidism in Tehran. Iran J Endocrinol Metabol, 2 ( 4): 263–77.

[16] Ko DH Jun SH Park KU Song SH Kim JQ Song J (2011). Newborn screening for galactosemia by a second-tier multiplex enzyme assay using UPLC-MS/MS in dried blood spots. J Inherit Metab Dis, 34: 409–414. 21340634 10.1007/s10545-011-9291-y

[17] Kingsmore SF Lantos JD Dinwiddie DL Miller NA Soden SE Farrow EG Saunders CJ (2012). Next-generation community genetics for low- and middle-income countries. Genome Med, 4 (3): 25. 22458566 10.1186/gm324 PMC3446275

[18] Groselj U Tansek MZ Smon A Angelkova N Anton D Baric I Djordjevic M (2014). Newborn screening in southeastern Europe. Mol Genet Metab, 113 ( 1–2): 42–5. 25174966 10.1016/j.ymgme.2014.07.020

[19] Vallian S (2009). Quantitative Bacterial Micro-Assay for Rapid Diagnosis of Galactosemia: Application in Galactosemia Neonatal Screening. Journal of Sciences, Islamic Republic of Iran, 20 ( 4): 319–23.

[20] Agarwal M Joshi K Bhatia V Gopalakrishnan V Dabadghao P Das V Pandey A Kumar M Phadke SR (2015). Feasibility study of an outreach program of newborn screening in Uttar Pradesh. Indian J Pediatr, 82 ( 5): 427–32. 25366286 10.1007/s12098-014-1557-6

[21] Gopalakrishnan V Joshi K Phadke S Dabadghao P Agarwal M Das V Jain S Gambhir S Gupta B Pandey A Kapoor D Kumar M Bhatia V (2014). Newborn screening for congenital hypothyroidism, galactosemia and biotinidase deficiency in Uttar Pradesh, India. Indian Pediatr, 51 ( 9): 701–5. 25228601 10.1007/s13312-014-0485-x

[22] Lund AM Hougaard DM Simonsen H Andresen BS Christensen M (2012). Biochemical screening of 504,049 newborns in Denmark, the Faroe Islands and Greenland--experience and development of a routine program for expanded newborn screening. Mol Genet Metab, 107 ( 3): 281–93. 22795865 10.1016/j.ymgme.2012.06.006

[23] Cuthbert C Klapper H Elsas L (2008). Diagnosis of inherited disorders of galactose metabolism. Curr Protoc Hum Genet, 17.5. 10.1002/0471142905.hg1705s56 18428423

[24] McCabe C (2009). What is cost–utility analysis? University of Leeds, England. Available from: http://www.bandolier.org.uk/painres/download/whatis/What_is_cost-util.pdf.

[25] Lee J Joseph R Rajadurai VS (2008). Neonatal Screening–A Global Perspective. Ann Acad Med Singapore, 37 ( 12 Suppl): 1–2. 19904442

[26] Fox-rushby J Cairns J (2005). Economic Evaluation. Reprint. McGraw-Hill Education, UK.

[27] Camelo Junior JS Fernandes MI Jorge SM Maciel LM Santos JL Camargo AS Jr Passador CS Camelo SH (2011). [Newborn screening for galactosemia: a health economics evaluation]. Cad Saude Publica, 27 ( 4): 666–76. 21603750 10.1590/s0102-311x2011000400006

[28] Dhondt JL (2010). Expanded newborn screening: social and ethical issues. J Inherit Metab Dis, 33 ( Suppl 2): S211–7. 10.1007/s10545-010-9138-y 20544288

[29] Rastgar M (2010). Neonatal screening. Shiraz University of Medical Sciences, Iran. Available from: http://fhc.sums.ac.ir/files/gh-vagir/screening.pdf.

[30] Central Bank of the Islamic Republic of Iran’s Web Site (2010). Available from: http://www.cbi.ir/exrates/rates_fa.aspx.

[31] Tiwana SK Rascati KL Park H (2012). Cost-effectiveness of expanded newborn screening in Texas. Value Health, 15 ( 5): 613–21. 22867769 10.1016/j.jval.2012.02.007

[32] Camelo JS Jr Fernandes MI Maciel LM Scrideli CA Santos JL (2009). Galactosaemia in a Brazilian population: High incidence and cost–benefit analysis. J Inherit Metab Dis, 32: 1: S141– 9. 19418240 10.1007/s10545-009-1112-1

[33] Carroll AE Downs SM (2006). Comprehensive Cost-Utility Analysis of Newborn Screening Strategies. Pediatrics, 117 ( 5): S287–95. 16735255 10.1542/peds.2005-2633H

[34] Norman R Haas M Wilcken B (2009). International perspectives on the cost-effectiveness of tandem mass spectrometry for rare metabolic conditions. Health Policy, 89 ( 3): 252–60. 18823674 10.1016/j.healthpol.2008.08.003

[35] Wilson JMG Jungner G (1968). Principles and practice of screening for disease. World Health Organization. Available from: http://apps.who.int/iris/bitstream/10665/37650/17/WHO_PHP_34.pdf.

[36] Padilla CD Lam ST (2008). Issues on Universal Screening for Galactosemia. Ann Acad Med Singapore, 37 ( 12): 39–3. 19904445

[37] Clague A Thomas A (2002). Neonatal biochemical screening for disease. Clin Chim Acta, 315 ( 1–2): 99–110. 11728413 10.1016/s0009-8981(01)00716-1

[38] Joshi SN Bayoumi R (2012). Newborn Screening Program for Oman: The Time is here and Now. Oman Med J, 27 ( 5): 346–347. 23074542 10.5001/omj.2012.89 PMC3472584

[39] Health Quality Ontario (2003). Neonatal screening of inborn errors of metabolism using tandem mass spectrometry: an evidence-based analysis. Ont Health Technol Assess Ser, 3 ( 3): 1–36. PMC3387775 23074443

[40] Hsieh SH Hsieh SL Chien YH Weng YC Hsu KP Chen CH Tu CM Wang Z Lai F (2010). Newborn Screening Healthcare Information System Based on Service-Oriented Architecture. J Med Syst, 34 ( 4): 519–530. 20703906 10.1007/s10916-009-9265-x

